# A systematic evaluation of explainable AI methods for high-dimensional transcriptome-based cancer survival prediction

**DOI:** 10.3389/fphys.2026.1830956

**Published:** 2026-04-22

**Authors:** Yiyi Zuo, Shuting Yang, Wenxue Zhao

**Affiliations:** 1 Shenzhen Campus of Sun Yat-sen University, Molecular Cancer Research Center, School of Medicine, Shenzhen, China; 2 Sun Yat-sen University Sixth Affiliated Hospital, Department of Neurosurgery, Graceland Medical Center, Guangzhou, China

**Keywords:** cancer, deep learning, explainable AI (XAI), survival prediction, transcriptomics

## Abstract

Explainable Artificial Intelligence (XAI) holds the promise to compensate for the “black-box” nature of deep learning which impedes transcriptome-based cancer survival prediction. However, there is a lack of systematic benchmarking XAI frameworks tailored for high-dimensional survival data. To bridge this gap, we systematically evaluated six representative XAI methods in three main categories: gradient-based, propagation-based, and perturbation-based approaches by using a Self-Normalizing Neural Network (SNN) as the baseline survival model. 6,248 samples across 15 cancer types from The Cancer Genome Atlas (TCGA) was analysed in this evaluation with a unified framework we developed. The evaluation metrics encompassed three key dimensions: prognostic factor enrichment (univariate Cox regression significance), biological consistency (supported by four authoritative databases, including OpenTargets), and explanation stability (Kuncheva Index). Among the six XAI methods, we find that DeepSHAP achieved the best overall performance, identifying the highest number of statistically significant prognostic factors while maintaining superior explanation stability; LRP (Layer-wise Relevance Propagation) showed slightly lower prognostic specificity but the highest consensus with biological databases in capturing general cancer genes, making it suitable for validating biological plausibility. In contrast, the perturbation-based method, PFI (Permutation Feature Importance) exhibited systematic failure and extremely low stability due to its inability to handle feature collinearity in high-dimensional transcriptomic data. Furthermore, we identified explanation stability as a robust proxy for the biological validity of the XAI. Collectively, **t**his study establishes an empirical framework for selecting trustworthy AI explanation tools for precision medicine.

## 1 Introduction

Cancer remains a leading cause of morbidity and mortality worldwide (Bray et al., 2024). While identification of molecular markers is crucial for precision oncology, the effective mining of these targets is impeded by the inherent challenges of high-throughput sequencing data due to high dimensionality, limited sample sizes, and significant tumor heterogeneity (Chen et al., 2021; Youngblood et al., 2021; Hou et al., 2025). Deep learning has demonstrated exceptional capacity for modeling complex non-linear interactions in transcriptomics, thereby promising significant advancements in survival prognosis and biomarker discovery (Tran et al., 2021; Unger and Kather, 2024; Cui et al., 2025; Yuan et al., 2025). However, its decision-making process remains inherently opaque. This “black-box” nature severely compromises its trustworthiness in clinical risk stratification and hinders the identification of reliable biomarkers (Jiang et al., 2020; Alleman et al., 2023; Zhang et al., 2025). Explainable AI (XAI) has emerged as an important bridge between computational predictions and biological insight. In biomedicine, *post-hoc* based XAIs have shown distinct capacity to generate explanation without altering the internal architecture (van der Velden et al., 2022; Malinverno et al., 2023; Toussaint et al., 2024). The prevalent XAIs are typically categorized into gradient-based (e.g., IG, GradientSHAP) (Novakovsky et al., 2023), propagation-based (e.g., LRP, DeepLIFT, DeepSHAP) (Chiaburu et al., 2026), and perturbation-based (e.g., PFI) approaches (Simic et al., 2025). The categorizing criterion is strictly based on their core attribution mechanisms: gradient-based methods rely on direct gradient calculation between inputs and outputs to realize feature attribution, while propagation-based methods achieve this via layer-wise decomposition of activation or output differences, and perturbation-based methods by artificially altering input feature values. DeepSHAP is classified as a propagation-based method because its core calculation is built on the layer-wise propagation of activation differences from DeepLIFT, with Shapley value approximation serving as a supplementary optimization rather than the core attribution mechanism.

Despite the potency of these XAIs, their reliability in survival prediction remains a subject of significant skepticism, owing to its low specificity (Qiu et al., 2025), compromised explanation stability (Yang et al., 2022). Also, the absence of rigorous selection criteria for specific architectures and data modalities frequently leads to discordant interpretations across studies. These limitations underscore the urgent need for a systematic evaluation of validity and performance of these methods. Recent initiatives have begun to benchmark various XAIs. For instance, Metsch et al. introduced the BenchXAI framework for breast cancer subtyping (Karagoz et al., 2026); Vidya et al. assessed fidelity and stability in fMRI data (Vidya et al., 2025). While these efforts have been relatively confined to single cancer types or binary classification tasks, there remains an absence of systematic benchmarking tailored for high-dimensional survival prediction across a pan-cancer landscape. Consequently, current frameworks fail to address the challenges of transcriptomic data, resulting in low effectiveness in cross-cancer interpretability standards.

**Figure 1:**
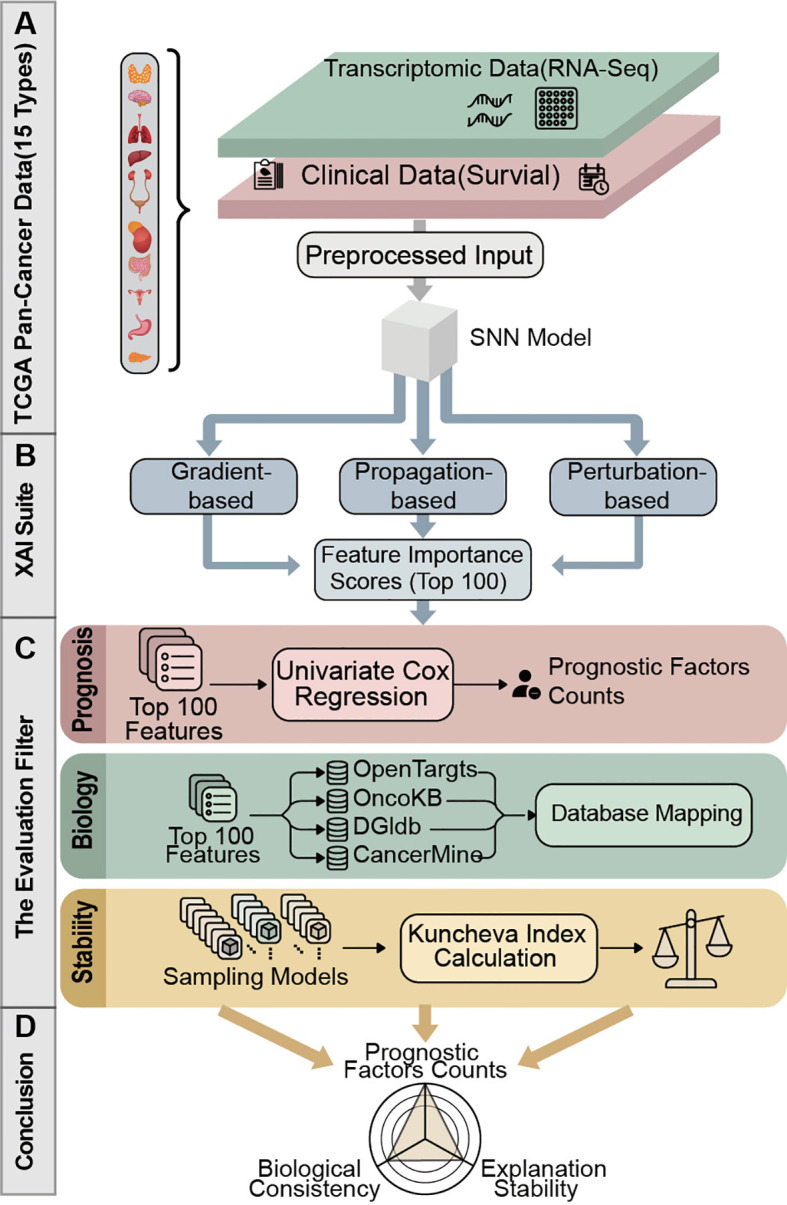
Schematic overview of the systematic evaluation framework for XAI for cancer survival prediction. **(A)** Transcriptomic data on 15 cancer types were collected and preprocessed to train SNN. **(B)** Trained SNN models were interpreted with three type algorithms to generate feature importance scores, isolating the top-100 most predictive features. **(C)** The reliability of interpretations was assessed via three distinct dimensions: prognosis factor counts, database mapping and stability. **(D)** Performance across these dimensions was synthesized to identify the premier XAI method.

To bridge this gap, we developed a unified interpretation framework leveraging 6,248 samples across 15 high-dimensional cancer transcriptomics by using self-normalizing neural network (SNN) as a robust baseline for survival modeling (**Figure 1**). We exclusively selected SNN as the baseline model for three key reasons: 1) SNN achieves self-normalization via SELU activation and Alpha Dropout, which effectively avoids gradient vanishing/exploding in high-dimensional transcriptomic data with low sample sizes; 2) SNN has been validated to outperform conventional deep learning models (e.g., MLP, CNN) in pan-cancer survival prediction with transcriptomic data, showing significant robustness; 3) A single and robust baseline model eliminates the interference of architecture differences on XAI performance evaluation, ensuring the fairness of cross-XAI comparison for our core research objective. we evaluated the six representative XAI algorithms aforementioned by systematically benchmarking their performance across three key dimensions: prognostic factor enrichment, explanation stability, and biological consistency. By doing so, we obtained empirical guidance to select optimal XAI tools to interpret model behavior. This work establishes a practical roadmap to navigate the trade-offs between algorithm reliability and data complexity.

## 2 Materials and methods

### 2.1 Dataset description

All preprocessed transcriptomic data and clinical information of pan-cancer patients were acquired via the UCSC Xena platform (UCSC Xena) at the University of California, Santa Cruz. In total, 15 cancer types and 6248 patient samples were involved for pan-cancer prognosis model training and testing, which range from 287–918 patients in individual cancers. The TCGA projects COAD and READ were combined into a single cohort, TCGA-COADREAD. We initially filtered genes using the Molecular Signatures Database (MSigDB) (Subramanian et al., 2005) and retained the top 2,000 genes accounting for 80% of the dataset variance as model inputs. Survival data, comprising overall survival time and censorship status, were formatted for discrete-time survival modeling.

### 2.2 Model construction

We employed a deep survival prediction framework based on SNNs selected for its demonstrated robustness in High-Dimension Low-Sample Size settings (Klambauer et al., 2017). Across the 15 TCGA cancer types, the SNN models demonstrated robust performance, achieving a median C-index of 0.622 (**Supplementary Figure 1**, **Supplementary Table 1**). The architecture comprises four consecutive SNN blocks. Each block consists of a fully connected layer, a Scaled Exponential Linear Unit (SELU) activation function and an Alpha Dropout layer (dropout=0.25). The hidden layers maintain a fixed width of 256 neurons. To be noted, we chose the SELU activation function because it induces self-normalizing properties in the network. Given that transcriptomic data is high-dimensional and can have varying scales, SELU helps maintain stable mean and variance of activations across layers, mitigating the vanishing/exploding gradient problem without the need for batch normalization, which can be unstable in small sample sizes. We specifically paired SELU with Alpha Dropout (as theoretically required by the SNN framework). Unlike standard dropout, Alpha Dropout maintains the self-normalizing property during training by using a multiplicative noise mask that preserves the mean and variance of the inputs, which is crucial for the robustness of the model in High-Dimension Low-Sample-Size (HDLSS) settings typical of cancer genomics. To optimize the model parameters, we use the Negative Log-Likelihood Loss function for a discrete survival model. We utilized the Adam optimizer (learning rate = 2×10^ (–4) with L1 regularization 1×10 ^ (–5) to induce sparsity and L2 regularization 1×10^ (–5) to enhance stability. We implemented an Early Stopping strategy augmented with a Warmup mechanism, defined by a 5-epoch warmup period and a minimum training cycle of 20 epochs. Training was terminated if the validation loss failed to improve for 7 consecutive epochs (patience). To ensure optimal model selection, we simultaneously maintained dual checkpoints corresponding to the lowest validation loss and highest validation C-index. During the final testing phase, the model achieving the maximal C-index was prioritized, with the minimal loss model serving as a fallback.

### 2.3 Experimental design and statistical framework

To rigorously benchmark XAI performance, we implemented a 10× repeated 5-fold Cross-Validation strategy for each cancer type, generating a total of 50 independent models per cohort. Within each fold, data were partitioned into an internal training set (80%) and an internal validation set (20%) for early stopping monitoring. Additionally, 100 Bootstrap samplings were applied to estimate confidence intervals for correlation analyses.

### 2.4 Interpretability framework

We evaluated six representative XAI algorithms spanning three primary attribution paradigms.

Gradient-based Methods:

1) Integrated Gradients (IG):Based on the axiomatic attribution theory, IG calculate the gradient path integral from the baseline (the mean of the training set) to the current input(implemented via Captum) (Sundararajan et al., 2017).

2) GradientSHAP: Combining the ideas of IG, SHAP, and SmoothGrad, the Shapley values are approximated by calculating the expected gradient on the background distribution of the training set (implemented via shap).

Propagation-based Methods:

3) DeepLIFT: The method addresses the issues of gradient saturation and loss of ReLU signals by decomposing the difference between the target output and the reference output (the mean of the training set) layer by layer to attribute the causes (implemented via Captum) (Shrikumar et al., 2017).

4) DeepSHAP:

This method combines the propagation efficiency of DeepLIFT with the game theory framework of SHAP. It modifies the DeepLIFT rules and estimates the expected value using Monte Carlo sampling and the entire training set (implemented via shap) (Lundberg and Lee, 2017).

5) Layer-wise Relevance Propagation (LRP):

We implemented LRP using the ϵ-rule (ϵ=1×10^(-6)) to ensure numerical stability. For linear layers, relevance was distributed proportional to weighted activations, excluding bias terms to maintain the conservation property. SELU and AlphaDropout layers were treated as identity pass-throughs for propagation (Paszke et al., 2019).

Perturbation-based Methods:

6) Permutation Feature Importance (PFI): This method measures the importance by randomly shuffling individual features and measuring the degree of decline in the model’s C-index. To reduce random errors, each feature is repeated for 5 times and the average value is taken (Fisher et al., 2019).

### 2.5 Evaluation metrics

Risk Score Calculation: To derive continuous risk scores from discrete-time outputs, we defined risk as the negative cumulative sum of survival probabilities over time. The calculation is formulated as Risk= -∑_t_S(t), where S(t) represents the predicted cumulative survival function. This metric serves as a negative proxy for expected survival time, where higher scores indicate shorter predicted survival.

Prognostic Factor Enrichment: To balance the inclusion of relevant biomarkers with the exclusion of noise, we focused on the top-100 features (k=100), because which was superior to k=50 or k=200 due to the “elbow point” of capturing signal Vs. noise. To validate the robustness of this threshold, we performed a sensitivity analysis across varying k values (k∈[20, 50, 100, 200, 300]), which confirmed that k=100 represents an optimal trade-off, capturing the core prognostic signals while excluding long-tail stochastic noise (**Supplementary Figure 2**). For the top-100 features identified by each model, we performed univariate Cox regression analysis using the lifelines library. Genes yielding a p< 0.05 were defined as statistically significant prognostic factors.

Biological Consistency: We mapped the top-100 features to four authoritative databases: OpenTargets, OncoKB, DGIdb, and CancerMine. This evaluation comprises three specific metrics:

1. The aggregate count of genes supported by the four databases.
2. The number of unique genes validated by at least one database.
3. The average number of database hits per validated gene, reflecting cross-database consensus.

### 2.6 Explanation stability

We employed the Kuncheva Index to quantify the overlap of the top 100 features across cross-validation folds. This metric corrects for chance agreement and ranges from -1 to 1, where higher values indicate superior stability of the attribution method.

### 2.7 Statistical analysis

Global differences were assessed using the Friedman test. *Post-hoc* pairwise comparisons were conducted using the Wilcoxon signed-rank test with Benjamini-Hochberg FDR correction for multiple hypothesis testing. Correlation analyses were evaluated using Spearman’s rank correlation coefficient. All statistical tests were two-sided, with significance defined as p< 0.05.

### 2.8 Software and environment

The entire analytical pipeline was executed in a Python (3.9.21) environment. The SNN models were developed using PyTorch (v[2.0.1]) with CUDA 11.7. Interpretability algorithms were deployed using the Captum (v[0.8.0]) library for IG and DeepLIFT, and the shap (v[0.48.0]) library for GradientSHAP and DeepSHAP.

To ensure temporal synchronization, four external knowledge bases database queries were performed on December 3, 2025. Specifically:

CancerMine: Literature-mined cancer gene associations (comprising 38,204 records) were retrieved from Zenodo (Record ID 16849846, version published August 13, 2025; https://zenodo.org/api/records/16849846/files/cancermine_collated.tsv/content).

OncoKB: Curated cancer genes were obtained via the official OncoKB API (v1) (https://www.oncokb.org/api/v1/utils/allCuratedGenes).

Open Targets: Gene-disease associations were queried using the Open Targets Platform GraphQL API (v4) (https://www.oncokb.org/cancer-genes).

DGIdb: Drug-gene interactions were downloaded from the Drug Gene Interaction Database official repository (https://dgidb.org/data/latest/interactions.tsv).

Code availability: The source code for training the TREE and reproduce the results is available on GitHub at https://github.com/ZYyli/xai-cancer-survival.git.

## 3 Results

### 3.1 DeepSHAP outperforms other XAI methods in discovery of prognostic factors from pan-cancer transcriptomic data

**Figure 2:**
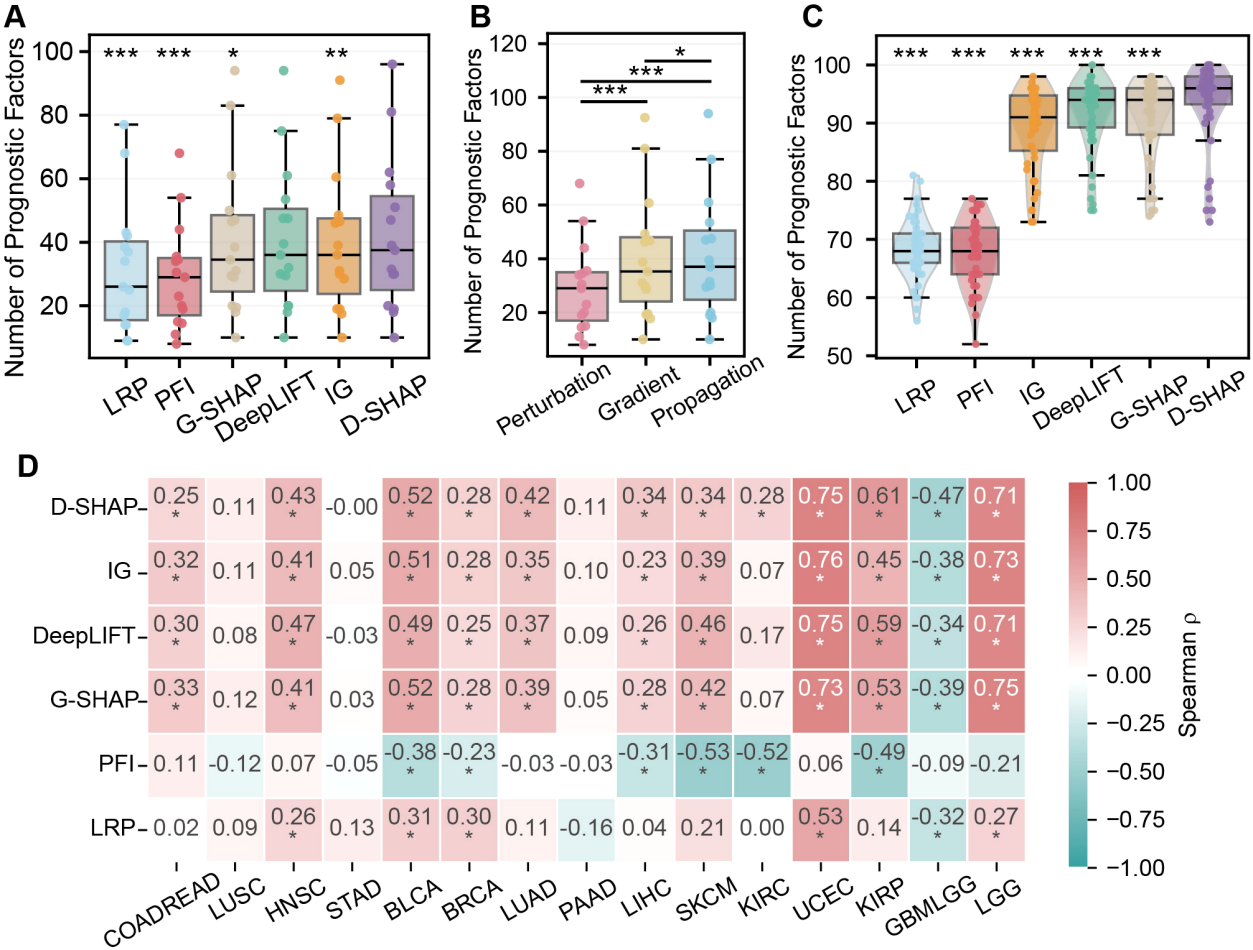
Systematic evaluation of prognostic factor counts across XAI methods. **(A)** Comparison of prognostic factor counts for six XAIs across 15 cancer types. **(B)** Comparison of prognostic factor counts for three XAI categories across 15 cancer types. **(C)** Detailed distribution for 50 models in LGG. **(D)** Heatmap displaying that spearman correlation analysis between model C-index and the number of prognostic factors. Cancer types are ordered from low (left) to high (right) C-index. Statistical Note: In box plots, the center line represents the median, box limits indicate the interquartile range (IQR), and whiskers extend to 1.5×*IQR*. In panels A and B, each cancer type was treated as an independent observational unit (n = 15) to avoid pseudoreplication at the patient level. Statistical significance across methods was first assessed using the Friedman test, followed by *post-hoc* pairwise comparisons using the Wilcoxon signed-rank test with multiple-testing correction by the Benjamini-Hochberg procedure. Methods are ordered from lowest (left) to highest (right) median value. In panels A and C, asterisks indicate comparisons between each method and the top-performing method. In panel B, asterisks indicate all significant pairwise comparisons. Full pairwise statistics are provided in **Supplementary Table 2**. Significance levels are denoted as *FDR *p* < 0.05, **FDR *p* < 0.01, ***FDR *p* < 0.001. Asterisks in panel D indicate statistical significance (*FDR *q* < 0.05).

To evaluate the clinical relevance of features prioritized by each XAI method, we quantified the number of statistically significant prognostic genes (univariate Cox regression, *p* < 0.05) among the top 100 ranked candidates. Across the pan-cancer cohort of 15 cancer types, we observed substantial difference in performance across methods (Friedman test, *p* < 0.001; Kendall’s *W* = 0.66; **Figure 2A**). Specifically, DeepSHAP consistently identified the strongest prognostic signal (median: 37.5; IQR: 29.5). In contrast, PFI and LRP detected markedly fewer prognostic genes than DeepSHAP (Wilcoxon signed-rank test, FDR-adjusted *p* < 0.001), with median counts reduced by 23.7% and 31.6%. These results suggest that DeepSHAP provides more clinically informative feature attribution in prognostic modeling. In terms of algorithm structure, propagation-based and gradient-based methods performed comparably and significantly outperformed perturbation-based approaches (Wilcoxon signed-rank test, FDR-adjusted *p* < 0.001, **Figure 2B**). This variability suggests that attribution strategies leveraging internal model structure are more effective at isolating clinically relevant signals from high-dimensional transcriptomics than strategies relying on external input perturbation.

Individual cancer type analysis revealed that DeepSHAP consistently ranked among the top-performing methods across most cohorts, including Glioblastoma and Lower Grade Glioma (GBMLGG), Brain Lower Grade Glioma (LGG), and Kidney Renal Papillary Cell Carcinoma (KIRP) (**Supplementary Figure 3**). For example, DeepSHAP not only enriched the highest number of prognostic factors (median: 96.0; IQR: 4.75) but also maintained a compact distribution across 50 sampled models in LGG (**Figure 2C**). In contrast, PFI and LRP showed fewer prognostic genes and showed greater variability, highlighting their limited robustness in capturing survival-associated signals.

We further examined the relationship between model concordance index (C-index) and prognostic factor counts (**Figure 2D**), with strong cancer-type dependency observed: cohorts with higher predictive performance (e.g., LGG, KIRP, Uterine Corpus Endometrial Carcinoma) showed robust positive correlations (Spearman’s *ρ*> 0.6, FDR-adjusted *q* < 0.05), indicating that well-performing models capture biologically meaningful prognostic information. Notably, GBMLGG exhibited a significant negative correlation, potentially indicating that predictive accuracy in this subtype may depend on complex non-linear representations beyond conventional univariate prognostic factors.

### 3.2 LRP achieves the highest biological consistency with established cancer gene databases

**Figure 3:**
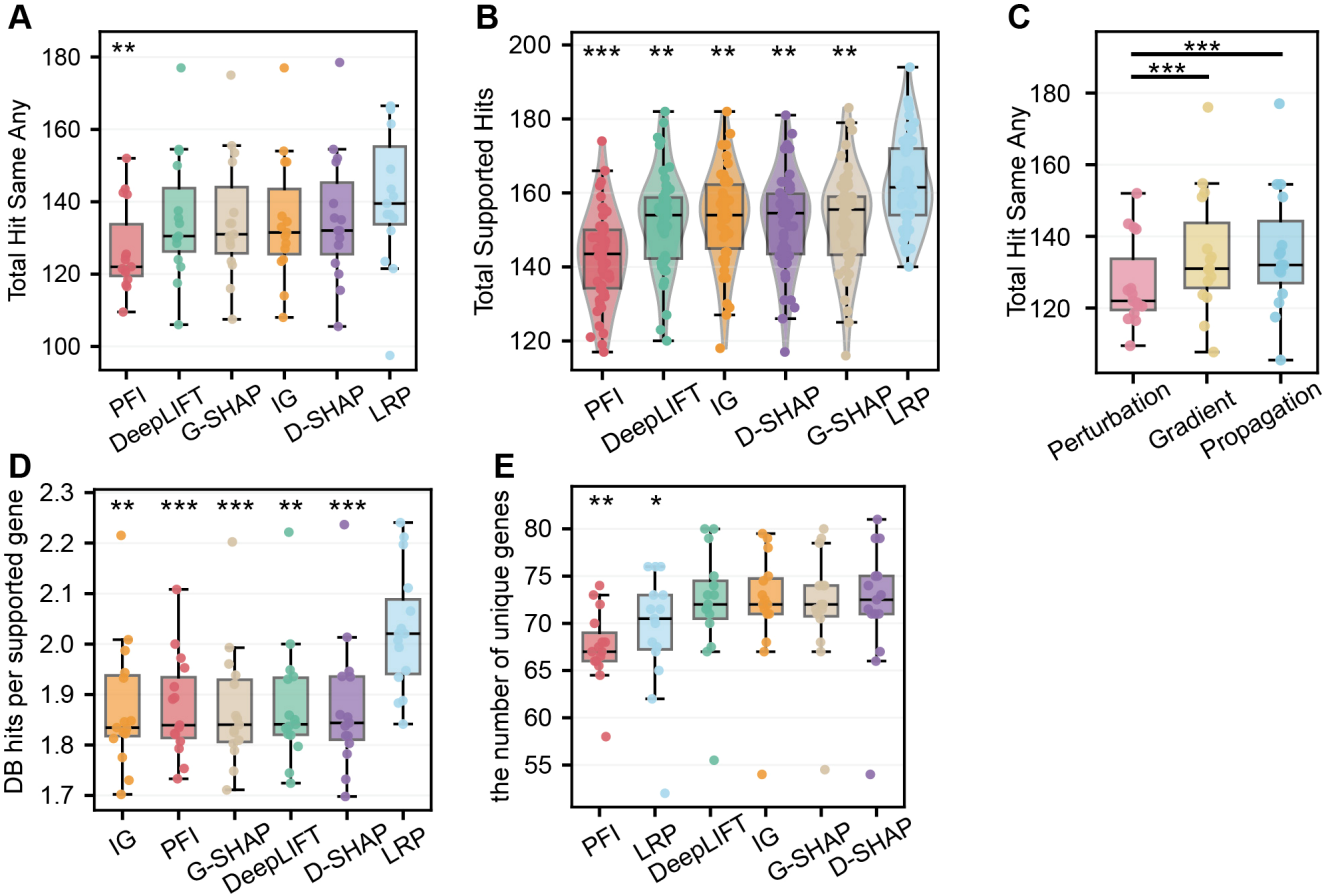
Benchmarking biological consistency using external knowledge bases. **(A)** Comparison of total hits in four databases for the top-100 features identified by six XAI methods across 15 cancer types. **(B)** Comparison of total hits in four databases for the top-100 features identified by three XAI categories across 15 cancer types. **(C)** Distribution of total supported hits for 50 models in Breast Invasive Carcinoma (BRCA). **(D, E)** Analysis of gene discovery preferences by DB hits per supported gene **(D)** and the number of unique genes **(E)**. Statistical tests and significance levels follow the conventions in **Figure 2**. Significance levels are denoted as *FDR p < 0.05, **FDR p < 0.01, ***FDR p < 0.001.

To assess the biological consistency of the identified features, we counted the number of top-100 genes mapped to four database (OpenTargets, OncoKB, DGIdb, CancerMine). Overall, we observed significant variation in gene retrieval performance across methods (Friedman test, *p* < 0.001, Kendall’s *W* = 0.34; **Figure 3A**). LRP achieved the highest level of database-supported evidence (median = 139.5; IQR = 21.5), whereas PFI showed the lowest evidence coverage (median = 122.0; IQR = 14.25; **Figure 3A**), indicating a relatively weak alignment with established biological knowledge. This trend was corroborated across the majority of cancer cohorts such as *BRCA* (**Figure 3B**). Categorically, both propagation-based and gradient-based methods significantly outperformed the perturbation-based method regarding database support (Wilcoxon signed-rank test, FDR-adjusted *p* < 0.001; **Figure 3C**). This pattern indicates that features highlighted by PFI may be more susceptible to stochastic noise rather than reflecting well-characterized cancer mechanisms. Interestingly, we found that LRP was able to identify more genes that across multiple databases (**Figure 3D**), but with less unique genes identified (**Figure 3E**), suggesting that LRP tends to recognize those common oncogenes that are well-known in the cancer researches.

### 3.3 Gradient- and propagation-based methods are markedly more stable than perturbation-based ones

**Figure 4:**
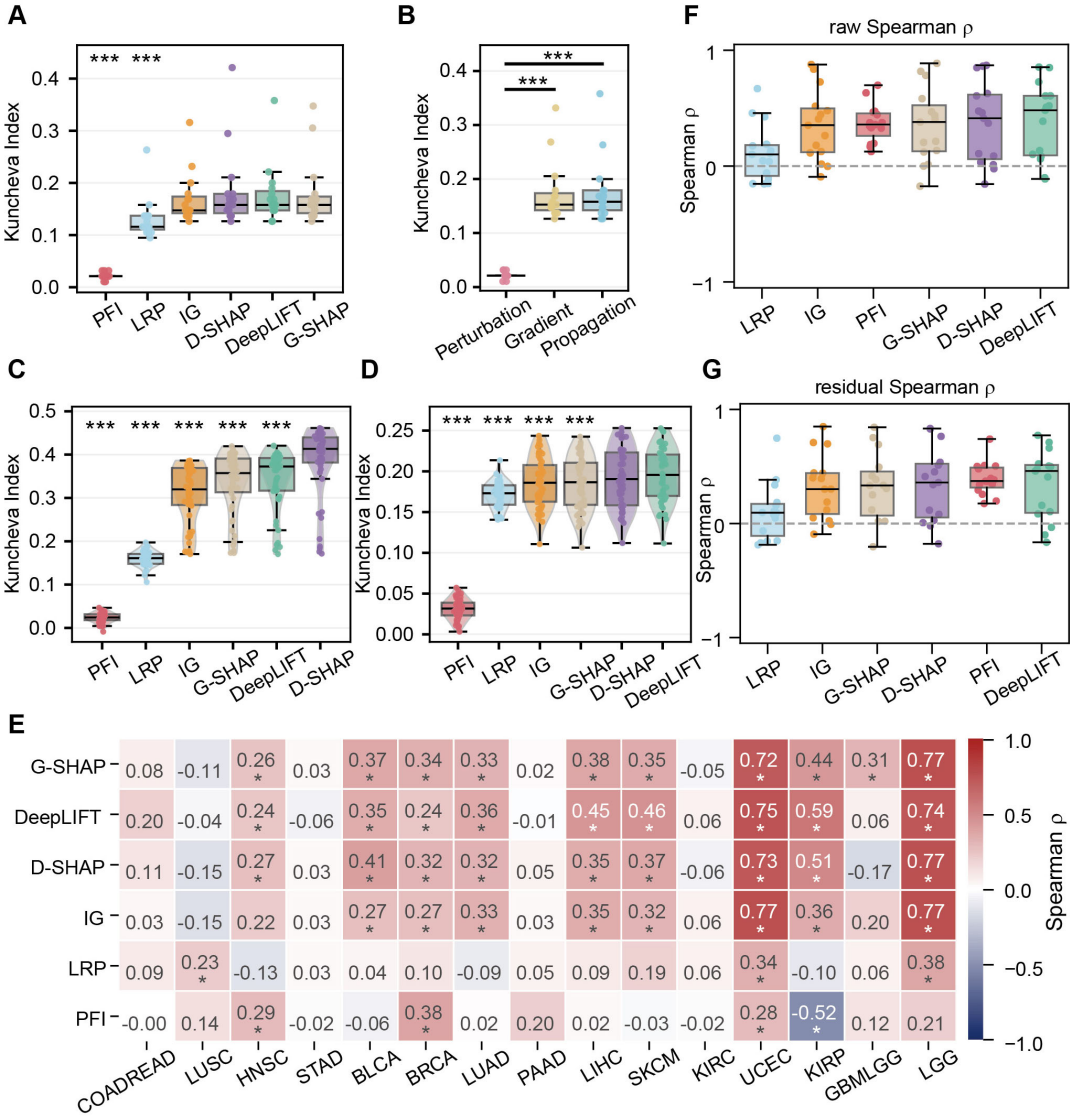
Evaluation of explanation stability and its association with model reliability. Stability is quantified via the Kuncheva Index, measuring the pairwise overlap of top-100 feature sets across different cross-validation folds within the same cancer cohort. **(A)** Comparison of explanation stability for six XAI methods across 15 cancer types. **(B)** Comparison of explanation stability for three XAI categories across 15 cancer types. **(C, D)** Detailed stability distribution for 50 models in LGG (left) and Liver Hepatocellular Carcinoma (LIHC) (right). **(E)** Heatmap displaying that spearman correlation analysis between model C-index and explanation stability. Cancer types are ordered from low (left) to high (right) C-index. Positive correlations (red) suggest that higher-performing models are predisposed to yielding more stable explanations. **(F, G)** Correlation analysis between prognostic factor counts and explanation stability by raw correlation **(F)** and by partial correlation **(G)**. Statistical Note: Statistical tests and significance levels follow the conventions in **Figure 2**. Significance levels are denoted as *FDR p < 0.05, **FDR p < 0.01, ***FDR p < 0.001. Asterisks in panel E indicate statistical significance (*FDR q < 0.05).

To evaluate explanation stability, we utilized the Kuncheva Index to quantify the reproducibility of the top 100 identified features across different cross-validation folds (10 repeats of 5-fold CV). Overall, gradient- and propagation-based methods exhibited significant higher stability across all 15 cancer types (Friedman test, *p* < 0.001, Kendall’s *W* = 0.82; **Figures 4A, B**); PFI had the lowest number (median ≈ 0.02; **Figures 4A, B**), indicating its limited ability to produce consistent explanations for high-dimensional transcriptomic data. However, to specific cancers, different XAIs showed different stability. Taking the LGG and LIHC for comparison, DeepSHAP achieved the most stable and compact distribution (median > 0.41) in LGG, While DeepSHAP and DeepLIFT achieved the highest index scores in LIHC (**Figures 4C, D**), suggesting selection of appropriate XAI methods is critical for translating explanation-derived biomarkers into clinically actionable insights.

To examine whether stability is indicative of validity, we investigated the relationship between model predictive performance (C-index) and explanation stability (Kuncheva Index) and found a clear cancer-type dependency (**Figure 4E**). In cohorts where the SNN achieved strong predictive performance (e.g., LGG and UCEC), explanation stability showed a strong and significant positive correlation with model accuracy (Spearman*ρ*>0.7, FDR-adjusted *q* < 0.05), suggesting that better-performing models tend to converge on more consistent feature subsets. In contrast, in cohorts with weaker predictive performance (e.g., COADREAD, LUSC, and STAD), the association was attenuated or not statistically significant. To further assess the biological relevance of stability, we correlated explanation stability with the number of prognostic factors (**Figure 4F**). Notably, even after adjusting for model performance using partial correlation analysis, stability remained a robust predictor of prognostic enrichment. We therefore concluded that stable attributions are more likely to reflect recurrent prognostic signals, whereas unstable explanations such as those produced by PFI may primarily capture stochastic variation rather than meaningful biology.

### 3.4 DeepSHAP delivers the optimal comprehensive performance across prognostic, biological and stability metrics

**Figure 5:**
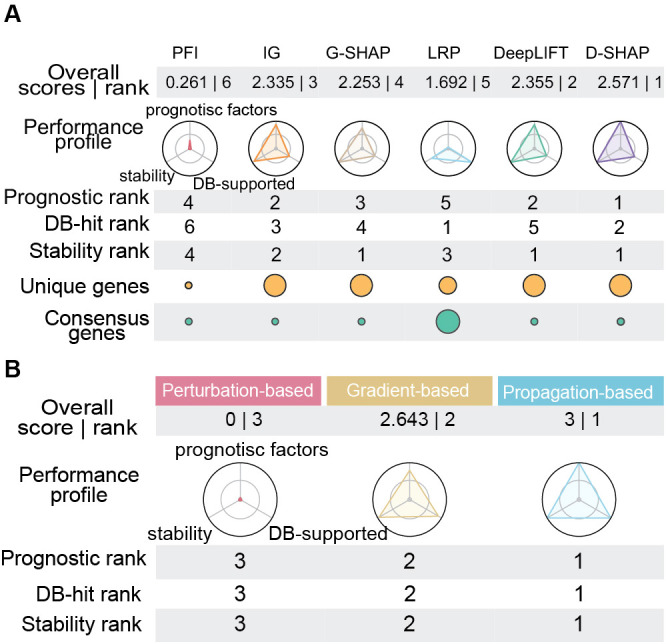
Comprehensive multi-metric benchmarking and ranking of XAI methods. **(A)** Global performance overview for the six evaluated XAI methods. Metric-specific rankings and bubble plots visualizing database discovery preferences. Yellow bubble size denotes the number of unique genes; Green bubble size denotes the average number of database hits per validated gene, reflecting cross-database consensus. **(B)** Global performance overview for three XAI categories. Overall Score and Rank, derived via Min-Max normalization of individual evaluation metrics; Performance profiles (radar charts) spanning three key dimensions: Prognostic Factor Counts, Explanation Stability, and DB-supported Hits. Larger polygon areas indicate superior and more balanced overall efficacy.

To develop a more comprehensive selection criterion, we incorporated three core dimensions (prognostic enrichment, biological consistency, and explanation stability) into a unified composite scoring framework using min-max normalization (**Figure 5A**). DeepSHAP attained the highest overall score of 2.571, showing the most balanced performance. Its radar profile was consistently strong, ranking first in prognostic factor enrichment and explanation stability, and second in biological consistency. Together, these findings highlight DeepSHAP as an optimal approach for prospective survival biomarker discovery.

In contrast, LRP showed a distinct performance profile. It ranked first in total database-supported hits, indicating strong alignment with well-established cancer consensus genes. However, its prognostic enrichment was comparatively limited (Ranked in top 5) and its stability was moderate (Ranked in top 3), resulting in an overall fifth-place ranking (score: 1.692) (**Figure 5A**). This suggests that LRP may be more appropriate for retrospective mechanistic validation rather than exploratory prognostic target identification. In the case of PFI, it showed consistently low performance across all dimensions, yielding the lowest composite score 0.261 (Ranked in top 6) and minimal coverage (**Figure 5A**). This further supports the limited applicability of perturbation-based attribution in high-dimensional omics settings. In terms of algorithm categories, the propagation-based methods ranked first in terms of score (Score 3.0), significantly outperforming the gradient-based methods (Score 2.643) and the perturbation-based methods (Score 0) (**Figure 5B**). This indicates that the propagation-based methods generally exhibit superior performance compared to the other two categories.

## 4 Discussion

High-dimensional transcriptomic profiling has become central to survival prediction in precision oncology. While deep learning architectures, particularly SNNs, have achieved notable performance improvements (Jiang et al., 2024; Waqas et al., 2025), their limited interpretability remains a major barrier to clinical adoption (Tun et al., 2025) To address this challenge, we performed a systematic benchmarking of six widely used XAI methods across a pan-cancer cohort spanning 15 cancer types, evaluating their prognostic relevance, biological consistency, and explanation stability. Our results highlight DeepSHAP as a particularly effective approach for interpreting survival models, demonstrating a favorable balance between identifying robust prognostic biomarkers and maintaining concordance with established biological knowledge. In contrast, perturbation-based methods such as PFI, although commonly applied in conventional tabular settings, showed limited reliability in high-dimensional genomic contexts, likely due to inherent methodological constraints. These findings provide practical guidance for selecting reliable attribution methods in transcriptomics-based prognostic modeling.

The systematic failure of PFI observed in our study highlights a pivotal challenge in applying XAI to high-dimensional omics data: the Curse of Dimensionality compounded by intricate feature collinearity. Gene expression profiles function inherently as dense co-expression networks rather than sets of independent variables (Dormann et al., 2013). By randomly permuting individual gene values, PFI inadvertently generates Out-of-Distribution (OOD) samples that violate these underlying biological correlation structures (Hooker et al., 2021; Molnar et al., 2024). This disruption forces the model to extrapolate into biologically implausible feature spaces, resulting in unreliable prediction shifts. Consequently, PFI exhibits negligible explanation stability (stability ≈ 0) and a marked deficiency in enriching for prognostic signals. It is worth emphasizing that gene expression is inherently a dense co-expression network. Unlike tabular data where features might be independent, genes function in pathways. Randomly permuting a single gene (as PFI does) creates biological implausibility because it breaks the co-regulation structure that deep learning models (like SNNs) learn. In high-dimensional spaces (thousands of genes), permutation forces the model to extrapolate into regions of the feature space that are never observed biologically (Out-of-Distribution). The instability (Kuncheva Index ~0) we observed is not just a mathematical artifact but a reflection of the model failing to make sense of biologically nonsensical inputs.

In contrast, propagation-based methods (e.g., DeepSHAP and DeepLIFT) mitigate this limitation by attributing the model output through backpropagation along the network structure, rather than relying on external feature perturbations. By following the internal architecture of the model, these approaches avoid generating biologically implausible inputs and yield more stable importance estimates. DeepSHAP further combines the efficiency of DeepLIFT with Shapley-based approximation principles, providing feature attributions that accurately quantify the marginal contribution of individual to the predicted survival risk while maintaining interpretability in high-dimensional transcriptomic settings.

Our findings underscore a critical divergence in the biological discovery patterns within propagation-based methods. LRP achieved the strongest validation performance, particularly in cross-database consensus, indicating a propensity to prioritize canonical cancer drivers genes that are extensively documented across multiple knowledge bases. However, while these well-characterized features serve as excellent indicators of biological validity, their ubiquity across general cancer populations often limits their discriminative resolution for precise patient risk stratification within specific cohorts.

In contrast, DeepSHAP exhibited greater prognostic specificity by isolating cohort-dependent drivers more directly associated with survival outcomes. This suggests that DeepSHAP is more sensitive to marginal gene contributions underlying inter-patient survival variability, making it particularly well suited for novel biomarker discovery. Accordingly, LRP may be best applied as a confirmatory sanity check of model alignment with established knowledge, whereas DeepSHAP is more appropriate for the prospective identification of clinically informative survival determinants. Those results provide practical guidance for selection of XAI methods in practice. For example, when exploring novel prognostic genes in understudied cancers (e.g., adrenocortical carcinoma), DeepSHAP is recommended for its superior enrichment of statistically significant features. While validating candidate genes against established pathways (e.g., confirming TP53-related signatures in ovarian cancer), LRP is preferred for higher consistency with KEGG/Reactome.

Finally, we uncovered a triadic link between model performance, prognostic enrichment, and explanation stability: high-performing survival models yielded more stable attributions, which were also more enriched for clinically meaningful prognostic signals. This suggests that explanation stability may serve not only as a technical reproducibility measure but also as a practical proxy for biological validity. When attribution methods capture genuine biological drivers, these features recur consistently across data splits, whereas instability-as observed with PFI-likely reflects noise-driven or spurious patterns. Therefore, Stability should be a prerequisite or a core secondary criterion. A model with a high C-index but low stability is likely overfitting to noise rather than learning robust biological signals, making Stability essential for clinical trustworthiness.

However, certain limitations exist in our study. First, our analysis was restricted to transcriptomic data. To validate the generalizability of our conclusions, future investigations should extend this evaluation framework to multi-omics landscapes. Second, we focused exclusively on the SNN architecture. While SNNs excel in high-dimensional tabular tasks, the interpretability dynamics of other emerging deep learning architectures, such as Transformers and Graph Neural Networks, warrant further investigation. Another key limitation of this study is that the analysis is restricted to transcriptomic data alone, while modern cancer survival prediction increasingly relies on multi-omics data integration (genomics, proteomics, epigenomics). Multi-omics data has the characteristics of higher dimensionality, significant inter-omics heterogeneity, diverse feature types and complex inter-omics correlation structures, and based on these characteristics, we reasonably speculate the generalization trend of XAI methods to multi-omics datasets: First, Propagation-based methods (DeepSHAP, LRP) will still be the optimal performers, as their layer-wise propagation mechanism has strong adaptability to high dimensionality and good compatibility with different feature types, which can effectively capture the nonlinear interaction between omics; Second, Gradient-based methods (IG, GradientSHAP) will experience performance decline due to more severe gradient saturation in ultra-high dimensional multi-omics data; Third, Perturbation-based methods (PFI) will still perform poorly, because the more significant collinearity and complex inter-omics correlation structures in multi-omics data will be severely damaged by PFI’s random permutation, generating more serious OOD samples. Future studies need to optimize XAI methods for multi-omics data characteristics (e.g., independent attribution and fusion of different omics features) to further improve their performance.

## Data Availability

The data presented in the study are deposited in the UCSC Xena repository. The RNA-seq datasets for the TCGA-COAD and TCGA-READ cohorts were obtained from the GDC Hub (https://xenabrowser.net/datapages/?dataset=TCGA-COAD.star_fpkm.tsv&host, https://xenabrowser.net/datapages/?dataset=TCGA-READ.star_fpkm.tsv&host). General access to other cancer datasets within the GDC hub is available at the UCSC Xena Browser (https://xenabrowser.net/datapages/). The original contributions presented in the study are included in the article/**Supplementary Material**. Further inquiries can be directed to the corresponding authors.
