# Supplementary material for: A systematic evaluation of explainable AI methods for high-dimensional transcriptome-based cancer survival prediction
Source: Front Physiol. 2026 Apr 22;17:1830956. doi: 10.3389/fphys.2026.1830956 (PMC13143651; doi:10.3389/fphys.2026.1830956)
Supplement: Supplementary file 1 [file Supplementaryfile1.docx]

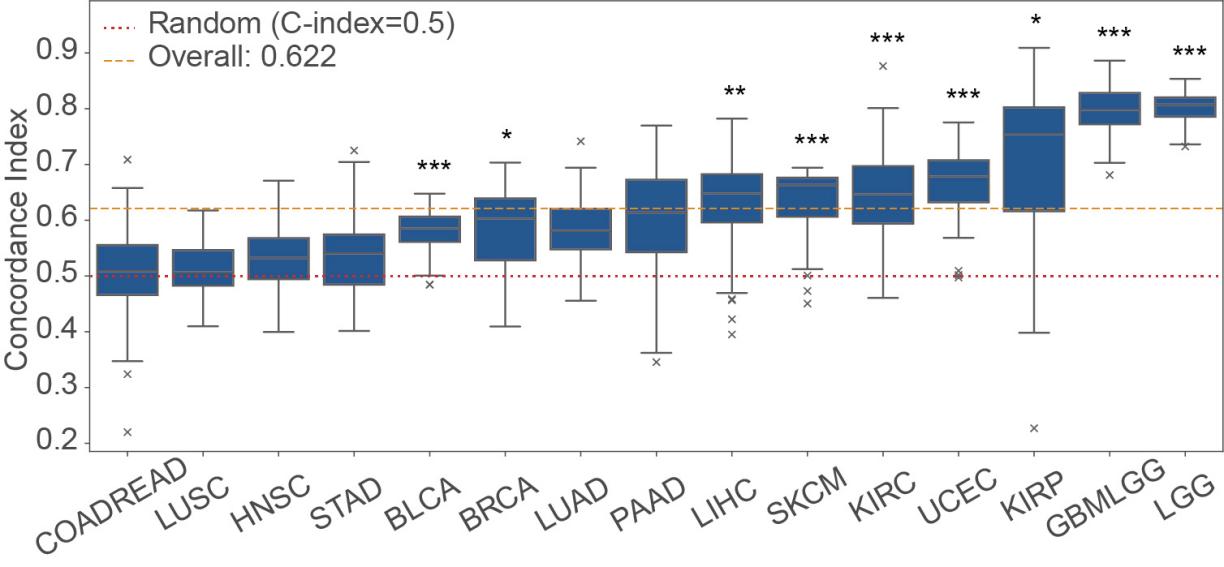


Supplementary Figure 1. Concordance Index Distribution for Different Cancers (Nested CV). The bar plots represent the mean values of five-fold cross-validation by SNN across 15 cancer types. * Represents significance of Kaplan-Meier analysis.


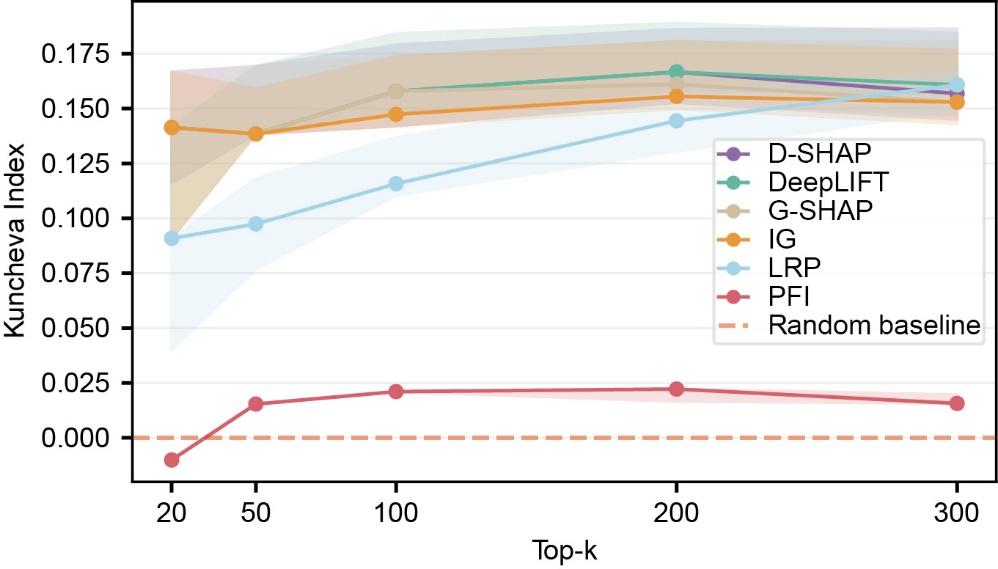


Supplementary Figure 2. Feature Stability vs Top-k by XAI Methods. From the initial set of 2000 candidate features, the top 20, 50, 100, 200, and 300 features were selected according to the importance ranking produced by each XAI method. The line plot illustrates how the feature stability of the six XAI methods changes as the number of selected top features increases.


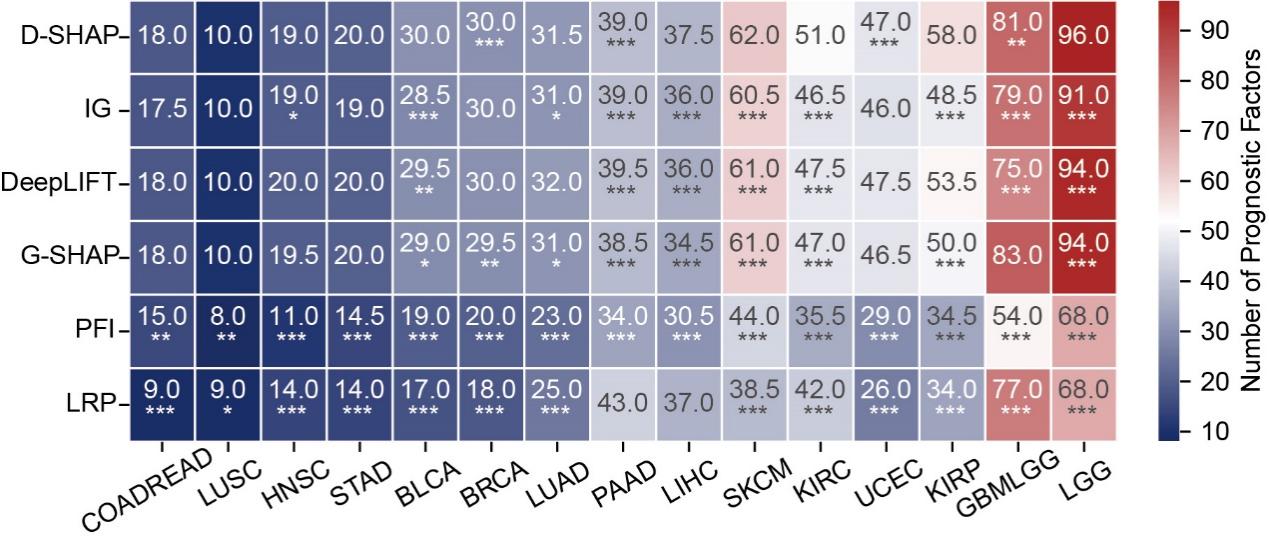


Supplementary Figure 3. Heatmap displaying the median number of prognostic factors for each method across 15 cancer types. Darker red indicates higher enrichment of prognostic signals. For each cancer, Statistical significance across methods was first assessed using the Friedman test, followed by post-hoc pairwise comparisons using the Wilcoxon signed-rank test with multiple-testing correction by the Benjamini-Hochberg procedure.Asterisks indicate significant differences compared to the top-performing group (* FDR p < 0.05, ** FDR p < 0.01, *** FDR p < 0.001)
