# Supplementary material for: A systematic evaluation of explainable AI methods for high-dimensional transcriptome-based cancer survival prediction
Source: Front Physiol. 2026 Apr 22;17:1830956. doi: 10.3389/fphys.2026.1830956 (PMC13143651; doi:10.3389/fphys.2026.1830956)
Supplement: Supplementary file 2 [file Table1.pdf]

Supplementary Table 1. C-index and AUC of SNN - based survival models for TCGA cancers

cancer_type	n_folds	integrated_logrank_p	mean_cindex	std_cindex	ci_lower_cindex	ci_upper_cindex	mean_auc	std_auc	ci_lower_auc	ci_upper_auc
BLCA	50	0.00044	0.57794	0.04193	0.56640	0.58930	0.57884	0.07654	0.55747	0.59934
BRCA	50	0.01256	0.57886	0.08034	0.55572	0.60136	0.48856	0.08966	0.46449	0.51330
COADREAD	50	0.41086	0.50527	0.08676	0.47973	0.52703	0.52498	0.11764	0.49074	0.55744
GBMLGG	50	0.00000	0.80077	0.04410	0.78883	0.81285	0.83184	0.06632	0.81321	0.85033
HNSC	50	0.77304	0.53181	0.06331	0.51311	0.54820	0.52559	0.09145	0.49890	0.54896
KIRC	50	0.00045	0.65278	0.07632	0.63154	0.67495	0.67649	0.08084	0.65293	0.70043
KIRP	50	0.03002	0.71132	0.14137	0.67123	0.74706	0.64061	0.13651	0.60386	0.67771
LGG	50	0.00000	0.80118	0.03068	0.79230	0.80892	0.77446	0.07963	0.75351	0.79780
LIHC	50	0.00274	0.62901	0.08766	0.60325	0.65089	0.60652	0.10854	0.57397	0.63625
LUAD	50	0.05410	0.58278	0.05722	0.56748	0.60013	0.55716	0.07268	0.53862	0.57776
LUSC	50	0.75989	0.50996	0.05122	0.49686	0.52424	0.48375	0.07745	0.46276	0.50416
PAAD	50	0.34935	0.60043	0.10532	0.57221	0.63026	0.63090	0.16202	0.58875	0.67423
SKCM	50	0.00000	0.63217	0.06455	0.61447	0.64956	0.65417	0.07337	0.63351	0.67409

Performance metrics include the mean and 95% confidence interval (CI) for the Concordance Index (C-index) and Area Under the ROC Curve (AUC) across 50 folds. The integrated log-rank p-value was employed to evaluate the statistical significance of the survival difference between high- and low-risk groups across the entire cohort.
